# Economic evaluation of a conditional cash transfer to retain women in the continuum of care during pregnancy, birth and the postnatal period in Kenya

**DOI:** 10.1371/journal.pgph.0000128

**Published:** 2022-03-07

**Authors:** Tom Palmer, Neha Batura, Jolene Skordis, Oliver Stirrup, Fedra Vanhuyse, Andrew Copas, Aloyce Odhiambo, Nicholas Ogendo, Sarah Dickin, Alex Mwaki, Hassan Haghparast-Bidgoli

**Affiliations:** 1 Institute for Global Health, University College London, London, United Kingdom; 2 Stockholm Environment Institute, Stockholm, Sweden; 3 Safe Water and AIDS Project (SWAP), Kisumu, Kenya; University at Buffalo, UNITED STATES

## Abstract

There is limited evidence on the cost and cost-effectiveness of cash transfer programmes to improve maternal and child health in Kenya and other sub-Saharan African countries. This article presents the economic evaluation results of the Afya trial, assessing the costs, cost-effectiveness and equity impact of a demand-side financing intervention that promotes utilisation of maternal health services in rural Kenya. The cost of implementing the Afya intervention was estimated from a provider perspective. Cost data were collected prospectively from all implementing and non-implementing partners, and from health service providers. Cost-efficiency was analysed using cost-transfer ratios and cost per mother enrolled into the intervention. Cost-effectiveness was assessed as cost per additional eligible antenatal care visit as a result of the intervention, when compared with standard care. The equity impact of the intervention was also assessed using a multidimensional poverty index (MPI). Programme cost per mother enrolled was International (INT)$313 of which INT$ 92 consisted of direct transfer payments, suggesting a cost transfer ratio of 2.4. Direct healthcare utilisation costs reflected a small proportion of total provider costs, amounting to INT$ 21,756. The total provider cost of the Afya intervention was INT$808,942. The provider cost per additional eligible ANC visit was INT$1,035. This is substantially higher than estimated annual health expenditure per capita at the county level of $INT61. MPI estimates suggest around 27.4% of participant households were multidimensionally poor. MPI quintiles did not significantly modify the intervention effect, suggesting the impact of the intervention did not differ by socioeconomic status. Based on the available evidence, it is not possible to conclude whether the Afya intervention was cost-effective. A simple comparison with current health expenditure in Siaya county suggests that the intervention as implemented is likely to be unaffordable. Consideration needs to be given to strengthening the supply-side of the cash transfer intervention before replication or uptake at scale.

## Background

Reports from Kenya suggest that facility delivery, antenatal care (ANC) attendance and postnatal care (PNC) attendance have fallen during the COVID-19 epidemic, while stillbirths have increased [1, 2]. Recent events notwithstanding, suboptimal levels of health care utilisation in Kenya result from a variety of demand- and supply-side barriers to care, including distance to facility [3], high direct and indirect costs of seeking care [4], information asymmetry [4, 5] and female disempowerment [6]. A 2017 enquiry into maternal deaths in Kenya found that the care of 92.4% of women who died was suboptimal, and that 72.7% of cases were associated with one or more patient and family factors, such as lack of transport, delay in reporting to a health facility and lack of ANC [7]. At the national level, 46.1% of women reported at least one barrier to accessing healthcare in 2014, and this proportion was associated with socioeconomic risk factors such as education and wealth [8]. This also applies to maternal and child health services. In 2014, only 57.6% of women in Kenya received the minimum of four ANC visits then recommended by the World Health Organization (WHO), while 61.7% of infants did not receive a PNC check-up in the first week after birth [8]. WHO guidelines recommend a visit between 48–72 hours after birth at a health facility, followed by at least two further visits [9].

Several interventions have been proposed to address both demand and supply-side barriers that lead to low levels of health service utilisation in low- and middle-income countries (LMICs). Supply-side interventions aim to improve the quality of healthcare offered, and include staff training, provision of supplies and results-based financing [10–12]. From 2016 in Kenya a supply-side intervention in the form of a results-based financing mechanism was scaled up nationally for selected maternal and child services [13].

In contrast, demand-side mechanisms aim to incentivise users to increase healthcare utilisation. A variety of demand-side financing mechanisms have been shown to increase ANC attendance and facility- based delivery in LMICs [14–16]. One such mechanism is the use of conditional cash transfers (CCTs), which aim to overcome financial barriers to accessesing health services, and which recent systematic reviews suggest have increased health service utilisation [17–19]. In eight South Asian and Latin American countries, Glassman et al. [18] found CCTs were associated with increased utilisation of ANC and increased delivery at health care facilities. Similarly, in a synthesis of seven systematic reviews, Hunter et al. [19] find that cash transfers and voucher programmes can lead to an increase in ANC and PNC utilisation, in addition to delivery at a facility. However, effectiveness evidence from seven programmes in Sub-Saharan African (SSA) countries is more limited, although it suggests that cash transfers may have a positive impact on maternal and child health care utilisation [17]. In Kenya, a recent study found maternity vouchers and CCTs to be highly effective in increasing facility delivery rates [20].

While evidence on the comparative cost and cost effectiveness of programs is generally considered key to guiding effective health systems design, there is very limited evidence overall on the cost-effectiveness of cash transfer and voucher programmes to improve maternal and child health in Kenya and other SSA countries. In Uganda, a scheme providing pregnant women with vouchers for transport and service provider payment for ANC, delivery and PNC conducted a cost-effectiveness analysis from the provider and societal perspectives, finding costs per disability-adjusted life year (DALY) averted of USD$302 and USD$338 respectively [21]. Only one known study analyses the cost-effectiveness of CCT interventions to improve maternal and child health: a recent study in Nigeria offered a cash payment of $14 for mothers attending at least three ANC visits, delivering in a health facility and attending at least one PNC visit [22]. They found that 26% of women did so in the intervention arm, compared with 12% in the control arm, leading to a 22% reduction in the stillbirth rate. The authors report payment of USD$143 in direct transfers per life saved, in this case through averted stillbirths. However, as a full program costing was not conducted, the authors assumed administrative costs were double the direct cost of incentives to derive an incremental cost-effectiveness ratio (ICER) of USD$429 per life saved. Finally, when also accounting for the direct cost of additional healthcare utilisation, they suggest an ICER of USD$693 per life saved. Although there is uncertainty in the total program implementation costs, this intervention is likely to be considered cost-effective under commonly used thresholds [23].

This article presents the economic evaluation results of the Afya trial, implemented in Siaya county, Kenya, between May 2017 and December 2019. The Afya intervention was a CCT aiming to retain women in the continuum of care, from their first ANC visit until their children reach 1 year of age [24]. In contrast to Okeke and Abubakar [22], the Afya intervention initiated transfers for each individual visit, rather than only for an entire package of care. Additionally, the Afya intervention disbursed payments via a mobile phone money transfer service, rather than through cash payments. The intervention had an impact on attendance of health care appointments, with a significantly higher proportion of appointments attended for eligible ANC (67% vs. 60%, adjusted OR (aOR) 1.90; 95% CI (1.36–2.66)) and child immunization visits (88 vs 85%; aOR 1.74; 95% CI 1.10–2.77) when compared with the control arm. The detailed results of the impact evaluation of the Afya intervention are published elsewhere [25]. Costs and cost-effectiveness of the demand-side financing intervention to improve maternal and child health outcomes in rural Kenya are presented in the present article. Additionally, by providing cost-efficiency estimates this study can offer insights into the optimal design of incentives to increase the utilisation of maternal and child health services in similar settings.

## Methods

### Ethics statement

The study received ethics approval from the Maseno University Ethics Review Committee, REF MSU/DRPI/MUERC/00294/16. Written informed consent was obtained from all participants. Details on consent to participation are provided in S1 Text. The trial was registered at the U.S. Government Clinical Trials website, ClinicalTrials.gov: NCT03021070.

### Study design, setting and population

A cluster-randomised controlled trial at 48 public health facilities was used to test the impact of the Afya intervention. Randomisation was done at the facility level, with only facilities offering full ANC profiling eligible for inclusion in the study. Women were recruited into the Afya trial at their first ANC visit. A policy mandating that public health facilities provide maternal health services at no cost to users was introduced by the Kenyan government in June 2013. Therefore, participants were not required to pay direct costs for their treatment. In order to participate in the study, women were also required to have mobile phone access and to have lived in their facility’s catchment area for at least 6 months. A total sample of 5,488 women were recruited into the trial. The intervention was implemented between May 2017 and December 2019.

The Afya intervention was implemented in Siaya County, Western Kenya, a county with human development and maternal and child health indicators below the national average [26]. Levels of MCH utilisation are historically low in Siaya county with only 18% of women reporting accessing all services along the continuum of care (ANC attendance, health facility delivery, PNC and newborn assessment) [27].

### Afya intervention

The Afya intervention provided a financial transfer to participating women for each verified visit they made to a heath facility for ANC, delivery, PNC and child immunisation. Women in the intervention arm received a transfer of KES 450 (around USD$4 in December 2020) per visit delivered through M-Pesa, a local mobile phone money transfer service. Women in the control arm were transferred a nominal gratuity of KES 50 (around USD$0.5) of mobile phone airtime, intended solely to incentivise the use of their study enrolment card if attending health facility appointments. All women participating in the study were given an enrolment card (henceforth Afya card) which was linked to a card reader device at their health facility. In addition to storing background characteristics of each woman, the Afya card was intended to automatically track each visit by touching on the card reader (similar to a contactless card payment). This would have initiated automatic payment of the CCT in the intervention arm, and of the airtime transfer in the control arm. However, the technology did not work as intended, meaning a large number of manual payment initiations were required. Manual payments were typically delayed, often by months. A full description of the study design, setting, intervention and implementation challenges is provided elsewhere [25, 28].

### Outcomes

Primary trial outcomes were proportion of eligible ANC appointments attended; proportion of eligible PNC appointments attended; proportion of eligible child immunization appointments attended and delivery at a health facility. Secondary trial outcomes were attendance of all eligible visits (both prenatal and postnatal), for each woman; the count of attended ANC and child immunisation clinic visits eligible for the primary outcome variables for each woman; the total number of ANC, child immunisation and PNC clinic visits (without applying any eligibility criteria) for each woman and gestational age at first ANC visit. ‘Eligible visits’ for the purpose of statistical analysis of the impact of the intervention were defined within a clear framework of expected visits but all scheduled ANC and PNC visits were eligible for payment. That is, additional conditions applied to the outcomes for the purposes of statistical analysis were not applied when initiating payments to participants [25]. Data on ANC visits were available for nearly all women involved, but data on other visit types could only be collected in a minority of women. However, the impact of this is limited to a degree by an analysis approach which models all outcomes simultaneously. All data analysed for the impact analysis were based on facility registers and clinic books carried by women to appointments. This overcame issues related to the Afya card not being used due to implementation challenges or women forgetting to bring the Afya card to appointments.

Outcomes in the intervention arm were compared with outcomes in the control arm to assess the impact of the cash transfer intervention. Although women in the control arm received a small airtime transfer, this was intended only to incentivise use of their Afya card. Therefore, outcomes are calculated with reference to KES 50 airtime transfer, which is considered equivalent to ‘current practice’. It was assumed that current practice was the same across intervention and control facilities. A detailed analysis plan for the Afya trial is reported elsewhere [24, 25].

While primary outcomes focus on proportions of each appointment type attended, corresponding secondary outcomes report visit counts and are used in the economic analysis to ease interpretation. When using full visit eligibility criteria for the analysis (i.e., only considering scheduled visits for ANC and capping child immunisation visits at four), the only statistically significant intervention effect on visit counts was for ANC clinic appointments. This represents the average additional number of eligible ANC visits per mother in the intervention arm compared to mothers in the control arm. No statistically significant difference in delivery at healthcare facility was found, and mortality data were not systematically collected. A full description of outcome measures and data sources is provided in the main trial article [25].

### Costing methods

A full methodology for the costing of the Afya intervention is provided in the trial economic evaluation protocol [29]. In summary, costs were estimated from a provider perspective, measuring economic costs to the implementing and non-implementing partners (programme costs), in addition to the direct costs of increased health service utilisation. All programme and provider costs can be considered incremental costs, reflecting costs in addition to the cost of standard care. Safe Water and AIDS Project (SWAP) in Kenya, was the implementing partner and the Stockholm Environment Institute (SEI) in Sweden, and University College London in UK were non-implementing or technical partners. Programme costs were sourced from financial project accounts of each institution, and included staff, transport (mainly to facilities for training, gadget delivery and troubleshooting), materials and capital costs. Key informant discussions guided conversion of financial costs to economic costs, which measure the opportunity cost of all resources utilised for intervention implementation, regardless of whether a financial cost was incurred. For example, such discussions guided estimation of current market value of capital items, particularly where no financial cost had been incurred during the project (e.g. for donated or existing items). Reported total costs include both start-up costs, such as community and health facility sensitisation, and implementation costs, such as the direct cost of transfers, in addition to staffing and system costs of initiating, validating and disbursing transfers.

Programme costs were collated in a customised MS Excel tool and a step-down costing methodology used to derive cost estimates [30]. Joint programme costs (i.e., those which could not be directly attributed to the intervention or other cost categories) were proportionally allocated to programme components on the basis of staff time-use data. Annualised capital costs were derived by assuming constant linear depreciation over the expected lifespan of the purchase. Research costs, as well as those related to implementing the airtime transfer in the control arm, were excluded from cost estimates and cost-effectiveness analyses, as they are not relevant components of either standard care or intervention scale-up. A proportion of monitoring and evaluation costs were allocated to the intervention to account for some element of each that is likely to be included in future implementation of similar interventions. Although monitoring and evaluation and quality assurance costs will be substantially less intensive at scale, it is likely that some element of each will still be required, particularly given potential implementation challenges. All programme costs were assessed over the full time horizon of the project (January 2016 –June 2020).

Additionally, costs incurred directly by the Ministry of Health in Kenya as a result of increased utilisation of health services attributed to the intervention (health service costs) over the implementation period were estimated. A facility costing questionnaire was used in each of the 48 participating facilities to collect data on expenditure, human resource use and service provision over the previous 12 months. These data were then supplemented with publicly available secondary data on drug prices and national procurement costs [31] to estimate unit costs of services provided by the facilities. Total health service provider costs include the additional cost from all visit types, including unscheduled visits, to account for the full burden on local health services.

Costs were estimated in Kenya Shillings (KES), or the local currency of each partner where relevant. Costs were inflated to 2020 base year values using local inflation rates and then converted to International Dollars, as is recommended for health care interventions where imported commodities do not account for a high proportion of costs (Turner et al., 2019). For example, costs within Kenya were converted to 2020 values using the Kenya consumer price index, which was most recently reported for 2018 [32]. An estimate for 2020 was therefore derived on the basis of recent annual growth rates. Finally costs were converted to International Dollars using purchasing-power parity conversion factors [33]. Costs incurred in other currencies (e.g., Swedish Krona) were converted into 2020 International Dollars in a similar way. Base-case estimates assume a 3% annual discount rate to convert to present values. All results are presented in both 2020 KES and INT$.

Total programme costs of the intervention from the provider perspective are presented, assuming that the cost of standard care is the same in both intervention and control arms. Average cost per mother was calculated by dividing the total cost of the intervention by the number of women enrolled in the intervention arm of the trial (i.e., intention to treat). The components of total cost were also summarised by category: staff costs, card reader system costs, direct costs of transfers and other costs (including capital and materials). A cost-efficiency indicator in the form of the cost transfer ratio was also calculated from a programme perspective. The cost transfer ratio represents the costs required to transfer one monetary unit to a recipient, excluding the direct cost of the transfer. Therefore, direct healthcare utilisation costs and patient costs are not included in these estimates. The cost transfer ratio was calculated as: *(Total programme costs-Direct cash transfer costs)/(Direct cash transfer costs)*.

### Cost-effectiveness analysis

Economic evaluation was conducted using the Afya trial results [25]. “Base case” ICERs are presented for additional eligible ANC visits and are calculated as the estimated additional cost of implementing the cash transfer alongside standard care, divided by the arithmetic mean difference in visit count outcomes between the intervention and control arms. Additionally, when not using eligibility criteria for the analysis, a statistically significant increase was also found for counts of PNC clinic appointments and child immunisation appointments. However, some of these visits would have been unscheduled and unpaid or surplus to the expected maximum required. The reasons for these increases are therefore more difficult to interpret and ICERs for this outcome are only reported in sensitivity analyses. Reported sensitivity analysis results for total visits counts use the sum of estimated incremental differences in mean visit counts as an alternative “base case” and use the upper and lower bounds of the 95% confidence interval for this sum for the minimum and maximum estimated ICERs. The confidence interval for the summed marginal difference in mean visit counts was calculated using the simplifying assumption of independence of the standard errors of the estimated marginal difference for each visit type. Outcomes were not discounted.

Further sensitivity analyses were conducted to explore the impact of uncertainty in costs and outcomes on cost-effectiveness results. Deterministic univariate sensitivity analysis was conducted by varying cost and outcome parameters between plausible minimum and maximum values. For eligible ANC visit counts, upper and lower bounds of the 95% confidence intervals were used to reflect uncertainty in intervention impact.

Plausible values for the cost transfer ratio were used to represent uncertainty in costs. Using data reported by the authors of a study assessing an unconditional cash transfer in Pakistan [34], a cost transfer ratio of 0.97 was derived when estimated from a programme perspective. Although this represents a substantial decrease in costs, this is perhaps plausible. For example, a prior cash transfer in Kenya targeting orphans and vulnerable children reduced its cash transfer ratio from 1.03 in a pilot to 0.34 following programme expansion [35]. Due to a lack of relevant plausible data from other interventions to inform the upper bound in sensitivity analysis, an arbitrary upper bound of +25% was assumed for the cost transfer ratio. Similarly, an assumed range of ±25% was used for direct health care utilisation costs, as such costs represent a very small proportion of total provider costs, and no plausible uncertainty range is clear based on available data. Finally, different discount rates were also used in the sensitivity analysis (i.e., 0% and 6%).

### Cost-effectiveness criteria

The outcomes used in this study are difficult to convert into standardised measures such as DALYs and quality-adjusted life-years (QALYs). Additionally, given the outcomes used, we are unable to use a well-established threshold to determine cost-effectiveness. We therefore present three estimates alongside our cost-effectiveness results to serve only an indicative purpose: national-level annual health expenditure per capita, county-level annual health expenditure per capita, and national level Gross Domestic Product (GDP) per capita. These estimates are presented to contextualise the magnitude of the cost of the intervention in this setting, rather than to be used as thresholds in determining whether the intervention is cost-effective.

### Equity impact analysis

The equity impact of the cash transfer intervention was explored by assessing how the impact of the intervention differed across socioeconomic groups. In addition to cost-effectiveness considerations, understanding the distribution of benefits is important as public spending should promote equity by improving the distribution of economic welfare [36]. This analysis provides useful information for decision-makers when considering any future-scale up of the intervention. Equity concerns can be explicitly considered within a multicriteria decision analysis approach to economic evaluation [37]. A multidimensional poverty index (MPI) was used to measure households’ socioeconomic status using the Alkire and Foster method [38]. A detailed description of the methods used to construct the index is provided in S2 Text. The proportion of households that are multidimensionally deprived, and that experience each individual deprivation, are reported, with confidence intervals accounting for clustering at the facility level.

MPI quintiles were used to analyse benefit incidence across socioeconomic groups. ANC clinic, PNC clinic and child immunisation visit counts were analysed using a multivariate Poisson mixed effects model, with random intercept terms at patient and the clinic levels. This corresponds to one of the models as described in the main trial impact analysis [25], with the addition of a factorial interaction of MPI quintile on outcome type and intervention effect. The marginal mean difference in visit counts between control and intervention clinics were calculated within each MPI quintile group.

## Results

### Costs

#### Programme costs

Table 1 summarises cost estimates for the Afya cash transfer intervention. The total programme costs were INT$787,187, of which the largest components were direct cash transfer costs (INT$231,915) and card reader system costs (INT$247,203). These two categories combined amount to 60% of total programme costs.

**Table 1:** Afya programme costs by component.

Cost component	Amount (2020 $INT)	Amount (2020 KES)	% of Total cost
**Primary cost components**			
Direct cash transfer costs (intervention arm)	231,915	9,651,199	29%
Card reader system costs	247,203	10,287,444	31%
Staff costs—Kenya	140,418	5,843,523	18%
Staff costs—Overseas	54,774	2,279,426	7%
**Other costs**			
Transport	32,150	1,337,917	4.1%
Training and sensitisation	6,245	259,892	0.8%
Nurse incentives	27,390	1,139,828	3.5%
Overheads	26,226	1,091,420	3.3%
Capital	1,088	45,267	0.1%
Miscellaneous*	19,779	823,123	2.5%
**Total programme costs**	**787,187**	**32,759,039**	**-**

$INT = International dollars, KES = Kenyan Shillings.

* Miscellaneous costs include costs such as server, postage costs, and other small cost items that could not be categorised.

Programme cost estimates in Table 2 suggest a cost transfer ratio of around 2.39. The total programme costs amount to INT$313 per mother, with around INT$92 of this comprising direct cash transfers.

**Table 2:** Cost-efficiency indicators.

Cost component	Amount (2020 $INT)	Amount (2020 KES)
Programme costs per mother (mean)	313	12,989
Direct cash transfer costs per mother (mean)	92	3,827
Cost transfer ratio	2.39	

$INT = International dollars, KES = Kenyan Shillings

#### Provider costs

Using data collected from the facilities, unit costs were calculated for each of the visit types that were statistically significant in the trial impact analysis: ANC visits (excluding first visit), PNC visits and child immunisation visits (Table 3). Unit costs were not calculated for facility delivery, as no statistically significant effect of the intervention was found for this outcome. Average estimated unit costs across facilities for ANC, PNC and immunisation visits were INT$4.97, INT$2.97 and INT$14.08 respectively. The differences in costs are largely explained by the different use of drugs and other consumables, particularly vaccines, reported for each of the visit types. Overall, direct healthcare utilisation costs reflected a small proportion of total provider costs, amounting to INT$21,756.

**Table 3:** Provider costs.

**Unit cost per visit type**	**Cost (2020 $INT)**	**Cost (2020 KES)**		**% Consumable cost**
Antenatal care visit	4.97	207		83%
Postnatal care visit	2.97	123		79%
Child immunisation visit	14.08	586		95%
**Total cost of additional healthcare utilisation**	**Cost (2020 $INT)**		**Cost (2020 KES)**	
Total additional visits per mother (ANC, PNC or immunization; no eligibility criteria applied)	21,756		905,365	
**Total provider costs**	**Cost (2020 $INT)**		**Cost (2020 KES)**	
Total cost	808,942		33,664,403	
Cost per mother	321		13,348	

$INT = International dollars, KES = Kenyan Shillings.

### Cost-effectiveness

#### Base case analysis

The incremental effect of the Afya cash transfer on ANC visits was 0.31 [0.15–0.47] when eligibility criteria are applied (Table 4). This results in a base case ICER of $INT1,035 per additional ANC visit. This is substantially higher than most recently available estimates for annual health expenditure per capita at the county and national levels, of $INT61 and $INT158 respectively [33, 39–41].

**Table 4:** Intervention outcomes and cost-effectiveness results.

Outcome	Average difference in mean [95% CI]	
Additional eligible ANC visits per mother	0.31 [0.15–0.47]	
	**Amount (2020 $INT)**	**Amount (2020 KES)**
Provider cost per additional eligible ANC visit	1,035	43,059
Siaya county annual health expenditure per capita, 2020/2021 [33, 39, 40]	61	2,538
Kenya health expenditure per capita, 2017 [33, 41]	158	6,349
Kenya GDP per capita, 2019 [42, 43]	4,509	185,270

$INT = International dollars, KES = Kenyan Shillings.Note: Currency conversions calculated based on relevant World Bank purchasing power parity conversion factors where required.

#### Sensitivity analysis

Results of univariate sensitivity analyses are summarised in Table 5, which shows the changes in ICER when cost components and outcomes are varied between their minimum and maximum plausible values as defined above. Sensitivity analysis is provided for both outcome variables. All estimated ICERs remain in excess of both national- and county-level health expenditure per capital (see Table 5).

**Table 5:** Sensitivity analysis results.

Parameter	Base case ($INT per additional visit)	Minimum ($INT per additional visit)	Maximum ($INT per additional visit)
Additional eligible ANC visits per mother	1,035	684	2,133
Discount rate	1,035	983	1,092
Cost transfer ratio	1,035	612	1,212
Direct healthcare utilisation costs	1,035	1,028	1,042
Total additional visits–no eligibility criteria applied*	245	175	423

$INT = International dollars, KES = Kenyan Shillings.

* This outcome includes visits for ANC, PNC or immunization. This parameter differs from other rows, which are all based on the main outcome for the economic evaluation, i.e., eligible ANC visit counts.

#### Equity analysis

Using the methodology outlined in the supporting information, results from the equity analysis suggest that 27.4% of participants’ household were multidimensionally poor (95% confidence intervals: 25.6%-29.2%). Amongst poor households, an average deprivation (or poverty gap) of 54.4% was estimated on average (i.e., each poor household suffered from 54.4% of measured deprivations, on average). This results in an adjusted poverty headcount (i.e., incidence of poverty weighted by the intensity of poverty) of 14.9%.

Table 6 shows a summary of all included indicators. Living standard is the dominant deprivation, followed by health and education. Although 28.0% of mothers had not completed primary school, only 11.7% of fathers had not completed primary school. This results in a low proportion of households (7.39%) deemed deprived in the education dimension, as OPHI methodology refers to the presence of *any* adult with at least 6 years education in the household.

**Table 6:** Indicators included in the multidimensional poverty index.

Indicator	Deprivation cut-off	Weight	Deprivation	Households Deprived % [95% CI]
**Education**				
Parental education	Mother education = “None” or “Primary incomplete” and Father education = “None” or “Primary incomplete”	1/3	Low education/low literacy	7.4 [6.6–8.17]
**Health**				
Child mortality	Did any of your children die before the age of 5 years? = “Yes”	1/3	History of child mortality in the household	21.4 [19.9–23.0]
**Living standards**				
Electricity	Electricity = “No” and Solar panel = “No”	1/21	No power source	51.3 [49.0–53.5]
Water	Water source = “Unprotected dug well” or “Unprotected spring” or “Surface water”	1/21	No safe water source	48.4 [41.8–54.9]
Sanitation	Toilet type = “Bucket/hanging toilet” or “Pit latrine without slab/open pit” or “No facility/bush/field”	1/21	Lack of access to private improved sanitation	69.2 [67.3–71.1]
Flooring	Floor = “Earth/sand” or “Dung”	1/21	Inadequate housing materials	67.0 [63.5–70.5]
Cooking Fuel	Cooking fuel = “Charcoal/coal/lignite” or “Wood/straw” or “Dung”	1/21	Household uses solid cooking fuel	95.1 [94.1–96.0]
Assets	Sum of clock = “yes”, radio = “yes”, television = “yes, phone = “yes”, fridge = “yes”<2	1/21	Low assets	27.4 [25.5–29.2]
Crowding	Number of household members per sleeping room > 3	1/21	Household is overcrowded	24.2 [21.8–26.5]
Overall proportion of multidimensionally poor households*				27.4 [25.7%-29.2%]

CI = confidence interval.

*A cut-off of 0.33 was applied identify “multidimensionally poor” households

**Fig 1:**
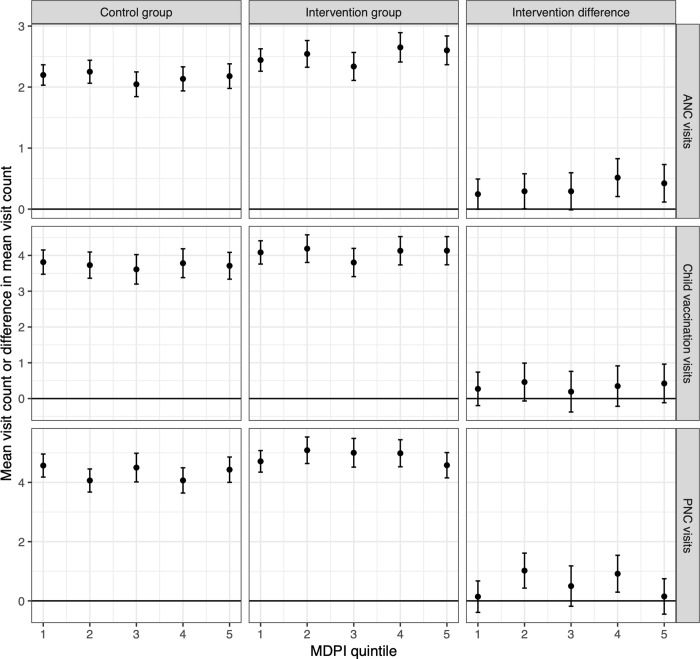
Outcomes by MPI quintile.

Fig 1 shows visit counts by each of the multidimensional deprivation quintiles constructed, displaying counts for each trial arm, in addition to the incremental difference (i.e., treatment effect). These were estimated among 4241 women included in the statistical analysis of primary and secondary outcomes for whom MPI values could be calculated. No clear trend by MPI quintile is apparent. Adding MPI interaction terms to the model analysing visit counts did not improve model fit, with a likelihood ratio test comparing the model with the simpler model suggesting an absence of statistically significant difference (p = 0.12).

## Discussion

This study contributes to the limited evidence on the cost-effectiveness of cash transfers to improve maternal, newborn & child health in Kenya and in other SSA countries. The total cost of designing and implementing the Afya intervention was INT$787,187, while an estimated additional INT$21,756 was incurred by health service providers as a result of increased utilisation following the intervention. A total of 2,522 women were enrolled into the intervention arm, resulting in a total provider cost of INT$321 per woman. An ICER of $INT1,035 per additional eligible ANC visit was estimated. Additionally, in the sensitivity analysis where eligibility criteria for the analysis were not used, an ICER of $INT245 per visit of any type was estimated.

It is not possible to conclude whether the Afya cash transfer intervention was cost-effective. This is largely due to the nature of the statistically significant outcome variables in this study (i.e., health facility visits, or fractions thereof), for which it is difficult to quantify clinical benefit in the absence of any collected morbidity outcomes. However, this study is in contrast to recent results from a similar cash transfer in Nigeria that found a significant reduction in child mortality [22]. Interestingly, the authors suggest that the key mechanism underlying this impact was increased ANC attendance.

A possible reason for the differences in outcomes is that gestational age at enrolment appears lower in the Nigerian trial, compared to in the Afya trial [27]. Delaying ANC initiation reduces the likelihood that pregnancy-related problems can be identified in a timely manner [44]. A variety of reasons have been proposed for late initiation of ANC. One potential reason is the cost of care-seeking [45]. Costs prior to confirmation of pregnancy may prove more of a barrier, as there is uncertainty with respect to the need to seek care. A study in Ethiopia found that time constraints, information constraints, distance to health facility and fear of long waiting time were all determinants of late initiation of ANC [46]. Pregnancy desire has also been found to be an important determinant of early ANC initiation [45, 47–50]. In contrast, unwanted pregnancies can result in late-care seeking due to delay in termination decision [51], intimate partner violence [52] or simply late recognition of pregnancy [53].

However, a systematic review of interventions to increase early ANC initiation suggests a lack of evidence on effective interventions to address this [54]. Conditional cash transfers like the Afya intervention are unlikely to address this important issue. One possible way of promoting earlier initiation of ANC is results-based financing. A systematic review of 11 countries predominantly in Sub-Saharan Africa highlights the potential of results-based financing to improve utilisation of MCH services [12]. Looking specifically at early ANC initiation, a results-based financing study in Zambia was found to reduce the timing of first ANC visit by two weeks [55]. Another study in Burkina Faso found that results-based financing increased the proportion of women attending their first ANC visit by 8.5 percentage points, while the overall number of advanced ANC visits increased by 27.7% [56]. However, the mechanisms through which this was achieved are not explained in that article, and the included districts were not randomly selected. A subsequent study found no clear impact of results-based financing on MCH indicators in Burkina Faso [57]. An evaluation of the impact results-based financing programmes have had on MCH utilisation in Kenya is ongoing [13].

The equity analysis highlights the extent of multidimensional poverty in this setting. Additionally, analysis of the impact of the intervention by MPI quintile highlights an important limitation of the study. Due to technology failures, payments were typically delayed, often until months after the visit [28]. For many participants, it therefore remains unclear to what extent financial barriers to care-seeking were removed by the intervention. Particularly for a poor household, if the transfer could not be relied upon to subsidise care-seeking costs, then it is not clear whether the intervention truly incentivised care-seeking.

While delayed payments have been documented in other cash transfer programmes, such as in Ghana [58], the impact of such delays on care-seeking behaviour remains uncertain. Although the design of the Nigerian study was different, paying the incentive only in one instalment after all conditions had been met, it is unclear whether this influenced observed differences. For example, infrequent payments can act as a commitment device [59]. Similarly, the impact of using cash in the Nigerian study compared to M-Pesa in Afya remains unclear.

Okeke and Abubakar [22] do not provide any actual estimates of programme costs for the Nigerian study, and instead rely on an assumed a cost transfer ratio of 2 for their cost-effectiveness analysis. As noted by Garcia and Saavedra [60] in a meta-analysis of 47 conditional cash transfer programs in low- and middle-income countries, there is a lack of data on administrative costs for most programs. The present study can therefore present some additional insight into administrative cost concerns for cash transfers to increase healthcare utilisation.

Although previously reported cost transfer ratios [34, 61] are lower than the cost transfer ratio of 2.4 estimated for the Afya intervention, in general, direct comparisons of cost transfer ratios are likely to be unreliable [35], given the differences in implementing organisation, sociocultural context, conditionality requirements, age of the program, intervention design and, perhaps most importantly, the value of the transfer. Considering these limitations, the absolute value of the transfer and the administrative cost per recipient should also be taken into account. For example, the NGO Give Directly provide a lump-sum unconditional cash transfer utilising M-Pesa in Kenya, with costs (2012 $USD) of around USD$99 per USD$1000 transfer [62]. Clearly, given the size of the transfer, the cost transfer ratio is substantially lower, but the administrative cost per household remains comparable to that estimated for the Afya intervention. This is despite an expectation that administrative costs are higher for a conditional cash transfer as compared to an unconditional transfer, due simply to the need to verify conditions have been met [63]. It is also worth noting that despite also using M-Pesa in Kenya, it appears that there remains a large amount of manual input required to verify and administer payments in this programme.

The cost-efficiency indicators discussed above are essential considerations for implementers and intervention scale-up. Siaya county annual health expenditure for the 2020/2021 financial year was estimated at around KES2.5 billion [40]. There were an estimated 28,260 births in Siaya county in 2019 [64]. The estimated cost per woman of the intervention was 13,348 (2020 KES). Scaling up to every pregnant woman in Siaya county would cost KES0.38 billion, or around 15% of total annual expenditure on health in the county. Although unit costs could be reduced as a result of economies of scale, based on the available evidence, the cost per additional visit of the Afya intervention likely remains too high. Any consideration of expanding the intervention must be on the basis of reliable card reader technology, that will substantially reduce the costs and increase the cost-efficiency of the cash transfer intervention.

### Limitations

This study has several limitations. First, we were unable to fully identify and measure all direct and indirect costs incurred by mothers as a result of the intervention. Similarly, as morbidity data were not collected, we were unable to quantify clinical benefit associated with the interventions. The main outcomes used in this economic evaluation are therefore not commonly used in cost-effectiveness analysis, and it was not possible to convert outcomes into, for example, disability-adjusted life years. Due to challenges with technology and acceptability of the card reader system, and the resultant mixed model of manual and card reader payments, it is difficult to accurately estimate what it would potentially cost to implement the intervention with a fully manual or fully automatic system. Such considerations are important both in terms of cost-efficiency and as they may change the impact of the intervention.

## Conclusion

This study provides estimates of the cost of implementing the Afya intervention in rural Kenya, contributing to limited overall evidence on the cost-effectiveness of cash transfer programmes to improve maternal and child health. Based on the available evidence, it is not possible to conclude whether the Afya intervention was cost-effective. A simple comparison with current health expenditure in Siaya county suggests that the intervention as implemented is likely to be unaffordable. Consideration needs to be given to strengthening the supply-side of the cash transfer intervention before replication or uptake at scale. Despite this, by providing a detailed analysis of costs to providers and the cost-efficiency of the intervention, this study contributes important evidence to inform the design of future cash transfer interventions to improve maternal and child health.

## Supporting information

S1 Text Full details on processes for informed consent to participation. (DOCX)

S2 Text Detailed description of multidimensional poverty index construction. (DOCX)

S3 Text Annualised cost data. (DOCX)
