# Supplementary material for: Economic evaluation of a conditional cash transfer to retain women in the continuum of care during pregnancy, birth and the postnatal period in Kenya
Source: PLOS Glob Public Health. 2022 Mar 7;2(3):e0000128. doi: 10.1371/journal.pgph.0000128 (PMC10021150; doi:10.1371/journal.pgph.0000128)
Supplement: S1 Text — (DOCX) [file pgph.0000128.s001.docx]

**S1 Text – Full details on processes for informed consent to participation**

Prior to enrolment into the study, participants were given information about the study by the enrolling facility staff. The information was shared verbally in the participant’s local language (Dholuo and Swahili), and augmented by an animated video produced for the study that was shown to eligible participants when they visited the clinic. All potential participants were informed that their participation in the study was voluntary, and that even after enrolment they could elect to withdraw from the study at any time they choose. Participants were also informed that their choice to participate or not participate in the study would not affect their access to health services or treatment, and that there was no penalty for not participating in the study. Those who agreed to participate signed the informed consent form. Study participants were given a take-home information sheet in the form of a calendar. The calendar containe contact information of the research team who they could call if they had any follow-up questions.
