# Supplementary material for: Economic evaluation of a conditional cash transfer to retain women in the continuum of care during pregnancy, birth and the postnatal period in Kenya
Source: PLOS Glob Public Health. 2022 Mar 7;2(3):e0000128. doi: 10.1371/journal.pgph.0000128 (PMC10021150; doi:10.1371/journal.pgph.0000128)
Supplement: S2 Text — (DOCX) [file pgph.0000128.s002.docx]

**S2 Text – Detailed description of multidimensional poverty index construction**

A multidimensional poverty index (MPI) was used to measure households’ socioeconomic status using the Alkire and Foster method (37). This process involves the following steps:

1. Constructing a set of indicators based on baseline household telephone survey data collection. This survey was intended to be conducted for all women following enrolment However, only 4313 out of a total of 5471 were interviewed.
2. Determining cut-off values for each indicator that determine whether a household is deprived in that indicator (see Column 2, Table 6).
3. Weighting within each dimension equally to derive an overall value for that dimension (see Column 3, Table 6).
4. Applying equal weights to each of the three core dimensions (education, health and living standards) to derive an overall index.
