# Supplementary material for: Economic evaluation of a conditional cash transfer to retain women in the continuum of care during pregnancy, birth and the postnatal period in Kenya
Source: PLOS Glob Public Health. 2022 Mar 7;2(3):e0000128. doi: 10.1371/journal.pgph.0000128 (PMC10021150; doi:10.1371/journal.pgph.0000128)
Supplement: S3 Text — (DOCX) [file pgph.0000128.s003.docx]

**S3 Text – Annualised cost data**

| **Afya annualised programme costs by component (2020 $INT)** | **Year** | | | | |
| --- | --- | --- | --- | --- | --- |
|  | 2016 | 2017 | 2018 | 2019 | 2020 |
| **Card reader system costs** | 153,169 | 79,620 | - | - | 14,415 |
| **Staff costs - Kenya** | 9,209 | 15,001 | 53,579 | 53,162 | 9,467 |
| **Other costs - Kenya** | | | | | |
| Nurse incentives | - | 3,458 | 18,111 | - | 5,820 |
| Training and sensitisation | - | 6,245 | - | - | - |
| Transport | 1,598 | 5,424 | 12,540 | 6,805 | 906 |
| Miscellaneous | - | 189 | 5,437 | 7,105 | 743 |
| Overheads | - | 97 | 1,685 | 3,245 | 659 |
| Capital | 177 | 197 | 469 | 205 | 40 |
| **Staff - Overseas** | 6,758 | 6,561 | 14,797 | 12,830 | 13,828 |
| **Other - Overseas** | | | | | |
| Transport | 1,332 | 226 | 3,223 | 96 | - |
| Miscellaneous | 680 | 2,062 | 80 | 2,009 | 1,476 |
| Overheads | 5,185 | 4,811 | 5,549 | 2,460 | 2,534 |
| **Direct cash transfer costs** | - | 10,442 | 90,804 | 84,602 | 46,068 |
